# Adams County Medical Society

**Published:** 1868-02

**Authors:** Joseph Robbins

**Affiliations:** Sec’y.


### ADAMS COUNTY MEDICAL SOCIETY.

The regular Quarterly Meeting of the Adams County Medical Society was held at the office of Dr. Robbins, Quincy, Ill., November 11, 1867, at 1½ o'clock P.M. The President, Dr. Louis Watson, in the chair.

*Present.*—Drs. Watson, Stahl, Cox, Helms, Kendall, Martin, Bassett, Bartlett, Ralston, Leach, Bane, Landon, Bonney, Wilson, and Robbins.

The minutes of the last Quarterly Meeting were read and approved.

Dr. Julius Guënther was proposed for membership by Dr. Wilson, and the proposition referred to the Censors.

On motion of Dr. Robbins, the Committee appointed, at the last Quarterly Meeting, to reïnvestigate the charges against Dr. Bassett and report at the next Annual Meeting, had leave to report at this meeting, and reported as follows:

"*To the Adams County Medical Society:*

"Your Committee, appointed at the Quarterly Meeting in August last, to reïnvestigate the charges against Dr. M. F. Bassett, which were heard and acted upon at the Annual Meeting, in May, 1867, beg leave to report that they have had the subject under consideration.

"Dr. Bassett appeared before the Committee and made a statement of the facts relating to the course which was made the basis of the charges, which statement is substantially embraced in the communication herewith presented and made a part of this report.

"Your Committee are satisfied that Dr. Bassett did not intend to set at defiance the Code of Ethics of the American Medical Association, which has been adopted as a part of the By-Laws of this Society; that if an unprofessional use was made of the circular letter which he designed for the medical profession alone, it was without his knowledge or consent, and to his deep regret; that he regrets that such a construction should have been put upon his letter, or his course, as to compromise, in any mind, the honor or dignity of the Adams County Medical Society, or the profession it represents; that, in view of the fact that the benefits which he hoped and believed would accrue to the profession and to suffering humanity may have been more than counterbalanced by the disaffection resulting from such misapprehension of his purpose and motives, he regrets that the circular letter was issued.

"The high moral and professional standing which Dr. Bassett had maintained since he became identified with this Society forbids, among those who know him best, the suspicion that he would be guilty of intentional quackery. From the frank and manly statements of Dr. Bassett, his bearing in our presence, and the uniformly scrupulous regard for the amenities and ethics of the profession which has ever characterized his professional intercourse with the various members of the Committee, we feel warranted in saying that there can be no doubt of his desire and intention to pursue such a course, hereafter, as shall give his professional brethren no occasion to feel either aggrieved or offended.

"We believe therefore, that professional harmony will be best maintained, and the dignity and honor of this Society and of the profession at large sufficiently vindicated, by meeting Dr. Bassett in the same spirit in which he has met your Committee. Respectfully submitted.

"I. T. Wilson,
M. M. Bane,
J. W. Bartlett,
Joseph Robbins."

Appended, is the communication of Dr. Bassett, referred to above:

"Quincy, Ill., Nov. 9, 1867.

"Dr. I. T. Wilson—*Dear Sir:*—At your request, I have the honor to communicate to you, in writing, the substance of the remarks which I made, last evening, before the Committee of the Adams County Medical Society having under consideration the charges pending against me, of having violated the Code of Ethics of the Society.

"It is a matter of serious regret that the course which was made the basis of those charges—including the publication of my circular letter to the profession, and the contents of the letter itself—should have subjected me to the suspicion of an intention to violate the ethics or compromise the dignity of the medical profession. While pursuing that course, I conscientiously endeavored to keep within the bounds of professional usage—never losing sight of my responsibility as a professional man—and if I transcended those bounds it was not my intention to do so. If an unprofessional use was made of my letter, it was without my consent and contrary to my wishes, and no one can regret it more than myself. My judgment approved the course which I pursued, but could I have foreseen that my motives would be misapprehended, and my professional brethren compromised by it, I should have avoided it.

"Respectfully, M. F. BASSETT."

One member of the Committee, Dr. Ralston, dissented from the report of the majority.

The report was received, and, on motion of Dr. Wilson, the case was dismissed by the following vote:

*Ayes*—Drs. Watson, Bartlett, Wilson, Robbins, Kendall, Helms, Cox, Bane, Landon, Bonney, Leach, Martin—12.

*Nays*—Ralston, Stahl—2.

The Censors reported Dr. Julius Guënther as having graduated at the New Orleans School of Medicine, his diploma bearing date April 15, 1857, and recommended his admission. On motion, he was regularly elected to membership.

Dr. Robbins proposed Dr. E. W. Goodwin for membership, which proposition was referred to the Censors.

The reading of essays being next in order, Dr. Landon, of Burton, read a highly interesting paper on the "Treatment of Acute Pneumonia." After some remarks on the paper by Dr. Ralston, and on his motion, Dr. Landon received the thanks of the Society for his essay.

Dr. Wilson, whose appointment as essayist was continued at the last Quarterly Meeting, was excused from further duty in that capacity, as he stated that the paper, which he then had prepared but was prevented by indisposition from reading, would have lost its interest, because the time had passed by when it was applicable.

Dr. Landon reported, verbally, three cases of paralysis complicating intermittent fever, in two of which the paralysis seemed to take the place of the agueish paroxysm, coming on in both cases at the time when the chill should have been expected. In the first case—hemiplegia—the paralysis still continues, after a lapse of two years. In the second—paraplegia—the patient recovered the use of his limbs in about six weeks, and at the time when the malarial cachexia seemed to be entirely removed. In the third case—hemiplegia—the paralysis followed a convulsion occurring during the first paroxysm of ague, and still continues. In all the cases the chills were easily interrupted.

Dr. Watson suggested that these might be cases of cerebro-spinal meningitis, which, as he had observed it, was generally attended with chills, though the periodicity was apt to be irregular.

Dr. Kendall regarded them as cases of congestion of the spinal cord, which might occur in intermittent, as in any other form of fever; and believed that cerebro-spinal meningitis was not a new or distinct disease, but a complication of fever, generally typhus.

Dr. Leach thought that Dr. Landon could not have been mistaken in his diagnosis. Like himself, Dr. Landon had encountered an epidemic of cerebro-spinal meningitis, in the same locality, and understood its characteristic features.

Dr. Stahl asked permission to withdraw from membership in the Society, saying that he had abandoned the practice of Medicine, and had no intention of resuming it; that he had ceased to read medical books, and did not feel like participating longer in the active business of the Society.

On motion of Dr. Ralston, Dr. Stahl was unanimously requested to retain his membership.

Dr. Stahl thanked the Society in a feeling manner for their kindness, but said that, for the reasons he had given, he felt that the time had come when he should lay off the harness.

Accordingly, on motion of Dr. Wilson, permission was granted him to withdraw.

The Society proceeded to the consideration of the charge preferred against Dr. E. D. Helms, at the last Quarterly Meeting, of having violated Article VIII of the By-Laws, by joining the so-called Quincy Medical Society, made up, in part, of expelled members of the Adams County Medical Society. The charge was read, and the Secretary reported that he had served Dr. Helms with a copy thereof, as directed by the Society.

Dr. Helms, being present, admitted that he had joined the so-called Quincy Medical Society, but claimed that it was not an infraction of any rule of this Society.

It becoming apparent that a full hearing of the case would protract the session to an hour unseasonable for members residing out of the city, it was ordered, on motion of Dr. Bassett, that the case of Dr. Helms, together with the case of Dr. Baker, against whom similar charges were pending, be referred to the Annual Meeting in May.

It was ordered that the proceedings of this meeting be published in the Chicago Medical Examiner and *Chicago Medical Journal.* Adjourned.

JOSEPH ROBBINS, M.D., *Sec'y.*

